# New Small Molecules Targeting Apoptosis and Cell Viability in Osteosarcoma

**DOI:** 10.1371/journal.pone.0129058

**Published:** 2015-06-03

**Authors:** Doris Maugg, Ina Rothenaigner, Kenji Schorpp, Harish Kumar Potukuchi, Eberhard Korsching, Daniel Baumhoer, Kamyar Hadian, Jan Smida, Michaela Nathrath

**Affiliations:** 1 Clinical Cooperation Group Osteosarcoma, Institute of Radiation Biology, Helmholtz Zentrum München—National Research Centre for Environmental Health, Neuherberg, Germany; 2 Department of Pediatrics and Children´s Cancer Research Center, Technische Universität München, Munich, Germany; 3 Department of Pediatric Oncology, Klinikum Kassel, Kassel, Germany; 4 Assay Development and Screening Platform, Institute for Molecular Toxicology and Pharmacology, Helmholtz Zentrum München—National Research Centre for Environmental Health, Neuherberg, Germany; 5 Lehrstuhl für Organische Chemie I and Catalysis Research Center (CRC), Technische Universität München, Garching, Germany; 6 Institute of Structural Biology, Helmholtz Zentrum München—National Research Centre for Environmental Health, Neuherberg, Germany; 7 Institute of Bioinformatics, University of Münster, Münster, Germany; 8 Bone Tumor Reference Center at the Institute of Pathology, University Hospital Basel, Basel, Switzerland; Faculté de médecine de Nantes, FRANCE

## Abstract

Despite the option of multimodal therapy in the treatment strategies of osteosarcoma (OS), the most common primary malignant bone tumor, the standard therapy has not changed over the last decades and still involves multidrug chemotherapy and radical surgery. Although successfully applied in many patients a large number of patients eventually develop recurrent or metastatic disease in which current therapeutic regimens often lack efficacy. Thus, new therapeutic strategies are urgently needed. In this study, we performed a phenotypic high-throughput screening campaign using a 25,000 small-molecule diversity library to identify new small molecules selectively targeting osteosarcoma cells. We could identify two new small molecules that specifically reduced cell viability in OS cell lines U2OS and HOS, but affected neither hepatocellular carcinoma cell line (HepG2) nor primary human osteoblasts (hOB). In addition, the two compounds induced caspase 3 and 7 activity in the U2OS cell line. Compared to conventional drugs generally used in OS treatment such as doxorubicin, we indeed observed a greater sensitivity of OS cell viability to the newly identified compounds compared to doxorubicin and staurosporine. The p53-negative OS cell line Saos-2 almost completely lacked sensitivity to compound treatment that could indicate a role of p53 in the drug response. Taken together, our data show potential implications for designing more efficient therapies in OS.

## Introduction

Osteosarcoma (OS) is an orphan disease with an incidence of approximately 0.4 per 100,000 population per year [1]. The rarity of the disease and thus the limited availability of biopsy material complicate the possibility of large-scale analyses of these tumors. In addition, the genetic complexity of OS up-to now hampered the identification of “druggable” OS-specific targets [2]. Although several genetic alterations have been described to occur in OS at varying frequency, OS are generally characterized by highly complex karyotypes [3,4], at least in a subset of tumors resulting from chromothripsis [5]. Thus, so far researchers have failed to identify an OS-specific mutation or a pathway.

These circumstances may partly explain that therapy in OS has not significantly improved in the last three decades. The standard therapy involves multidrug chemotherapy (methotrexate, doxorubicin, cisplatin and ifosfamide) in combination with radical surgery [6]. This treatment yields positive outcomes in many patients with an overall 5-year survival rate of approximately 70%. However, the prognosis drastically worsens in patients with apparent metastatic spread or recurrent disease with overall survival rates generally below 20% [7].

In the last years a great effort has been done to develop new therapeutic strategies for OS patients. Several studies and clinical trials have been testing multimodal therapies and dose variations of classical drugs. However, a great majority of these studies fail to enter phase III clinical trials [8]. Beside the challenge of establishing an adequate trial design in such a rare disease due to the lack of resources and the limited number of patients fulfilling all requirements, several studies had to be stopped due to low efficacy and safety [8,9].

Most molecular biology studies have focused on drugs that target single alterations associated with OS with the attempt to develop personalized therapies. Among the most promising drugs are small molecule kinase inhibitors, for example the inhibitor of insulin-like growth factor 1 receptor (IGF-1R) belonging to the family of receptor tyrosine kinases (RTK) [10], as well as p53-interacting drugs [11]. Insulin-like growth factors 1 (IGF1) and 2 (IGF2) stimulate certain pathways via the IGF receptor regulating cell growth and survival, pathways that are frequently deregulated in OS [10,12].

Likewise, *p53* mutations were associated with genomic instability in OS [13]. However in most of the tumors so far no loss-of-function mutations have been observed in the p53 protein. Consequently, the reactivation of the tumor suppressor function of p53 by the nutlin small molecule inhibitors of MDM2-p53 interaction seem very promising [11]. Recently, structural variations in intron 1 of the *TP53* gene were reported that could explain the absence of general p53 protein mutations but rather the altered activity of the p53 in OS [14,15]. Thus alterations in p53 binding affinity may cause the activation of different pathways that could contribute to malignant transformation [16]. Problems arising from the targeted therapies include amongst others the development of resistance, which is true for some RTK inhibitors [17], and the lack of predictive biomarkers to validate a positive outcome in patients [10]. Likewise, some drugs simply fail due to improper preclinical target validation and not lastly by the limited number of patients in which the respective target has been revealed [9].

Consequently, phenotypic drugs that target OS cell proliferation or metastasis are subjects of great interest in the development of new drugs [2,8,18]. Although in the past target-based approaches have been the gold standard in small-molecule high-throughput screens focusing on compound-target interactions [11,19], within the last decade more and more phenotypic screenings have been applied to discover small molecule inhibitors with new mechanisms of action [18,20–22].

Besides the discovery of new molecules from e.g. natural sources handled at the *in vitro* level, other promising agents such as maltonis are currently tested in phase I/II [23]. Still, the only new therapeutic agent in the treatment of OS that has been clinically approved in the recent years is muramyl tripeptide (MTP), an immunomodulatory drug [24]. Consequently, there is still a need for compounds from new sources and of diverse structural characteristics that would offer manipulation capability and could be tested in drug discovery evaluation processes.

In this study, we describe a phenotypic screening strategy that enabled us to screen a large set of diverse compounds (25,000) with regard to their effectiveness in OS cell lines. The step-by-step strategy allowed us to reduce the number of eligible compounds to a manageable number of compounds. We used this strategy to investigate compound actions with regard to their selectivity and apoptosis inducing potential in OS cells and compared these effects to well-known drugs that are used in clinical OS treatment.

## Results

### Screening strategy and compound identification

**Fig 1:**
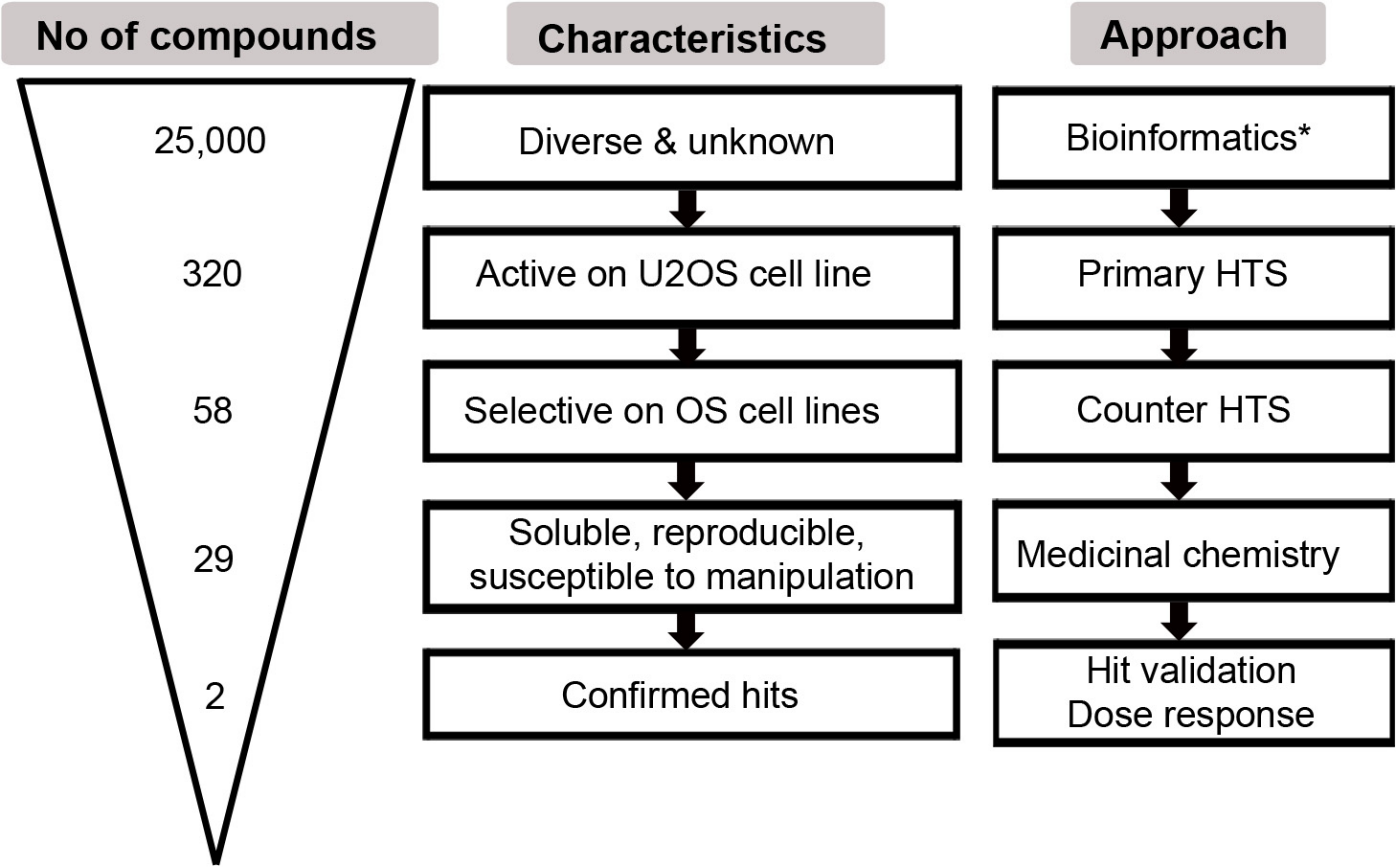
Workflow of the screening strategy. The graphic shows the number of compounds (triangle on the left), their characteristics (mid row) and the respective approach (right) that was applied for compound selection. Arrows from top to bottom indicate the “timeline“. *described in [19].

**Fig 2:**
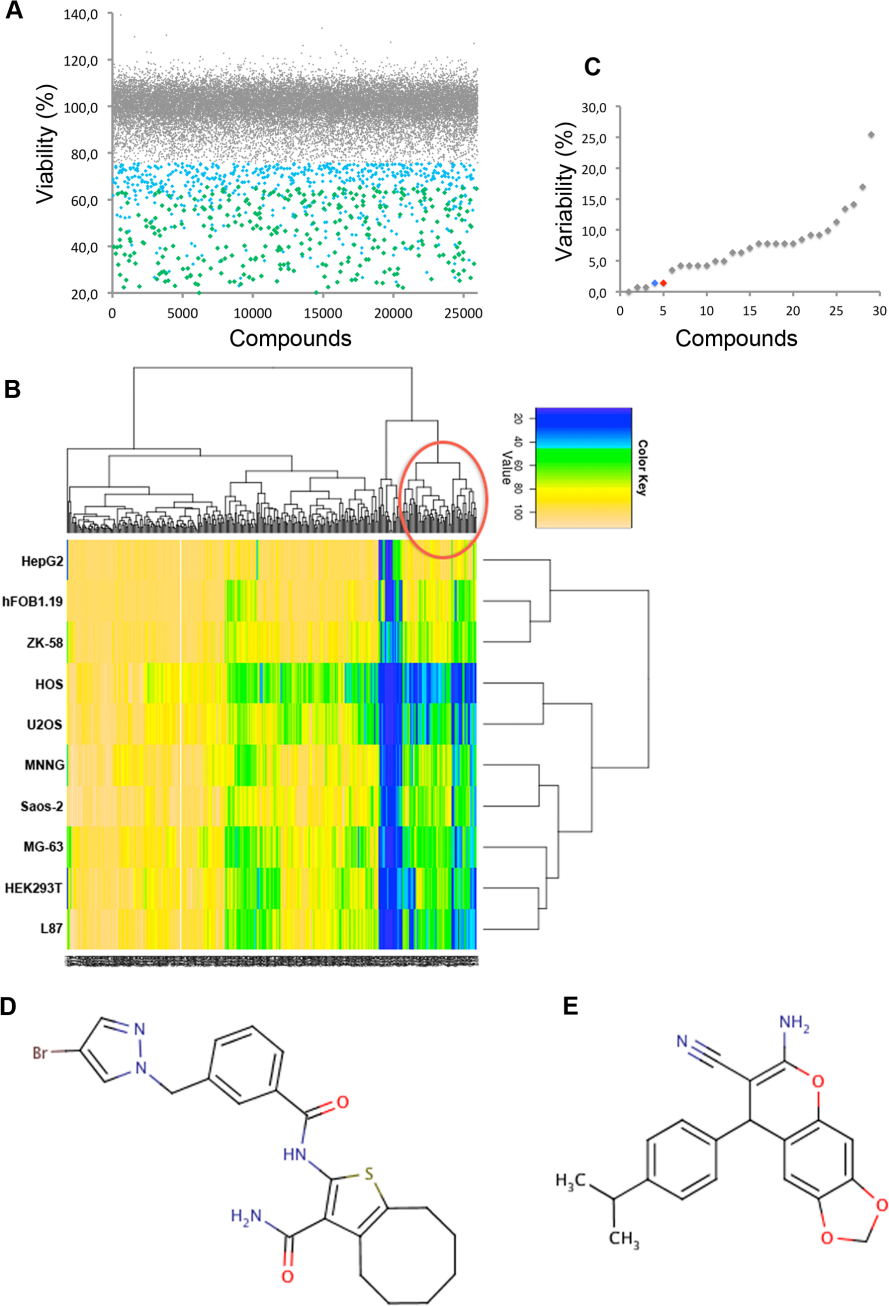
Compound selection process and compound chemical structures. A) Scatter plot shows distribution of 25,000 compounds in regard to cell viability (%) in U2OS cell line with compounds not significantly affecting viability (grey dots), those that reduced viability from 75 to 20% (blue dots), and defined active compounds that were selected for further analysis with viability ranging from 20 to 65% (green dots). Cell viability was determined using Celltiter Blue (CtB) assay. B) Hierarchical clustering of 320 defined active compounds. Compounds were screened on various OS and control cell lines. Respective cell lines are indicated on the left, color key shows values according to cell viability ranging from 20–100%. The red circle marks the cluster that was short-listed for subsequent medicinal chemistry analysis. C) The graph shows inter-replicate variability in the primary and the counter screen of the 29 short-listed compounds. A low inter-replicate variability was used as a selection criteria in medicinal chemistry analysis. The two confirmed hits are highlighted in red and blue, respectively. D) and E) show structures of the two confirmed OS-selective hits, namely compounds A13 (D) and H12 (E).

In order to identify new compounds that specifically target OS cells we screened three diversity libraries obtained from different suppliers (see Materials and Methods) that altogether comprised 25,000 diverse compounds. Fig 1 describes the workflow of the screening strategy used for compound identification. In a primary screen, the effects of compounds on cell viability were assessed in U2OS cells, a well-characterized OS cell line [25], using Celltiter Blue (CtB) assay (Fig 1, step 2). The primary screen at a final concentration of 10 μM resulted in 320 positive hits that reduced the cell viability of U2OS cells from 65 to 20% (Fig 2A). To identify those compounds that selectively act on OS cells, compounds were counter-screened in different OS and non-OS control cells (**Table 1**) using again cell viability as read-out (Fig 1, step 3). Here we used various well-known OS cell lines, including ZK-58 and MNNG-HOS as well as immortalized human osteoblasts hFOB1.19 [25,26]. From these results we performed a hierarchical clustering according to cell type and compounds with regard to their effects on cell viability. Clustering resulted in two main groups separating compounds with weak or no effects from those with strong effects on cell viability (Fig 2B). One group highlighted by a red circle could be further sub-divided into the compounds that generally reduced viability in all cell lines and those with the highest effects specifically in OS cell lines (Fig 2B, red circle). This latter subgroup consisting of 58 compounds specific to OS cell lines was further short-listed based on Lipinski´s rules on their medicinally relevant physical chemical properties [27]. The criteria included the solubility, cell permeability, assay reproducibility but also the ease of synthesis and the possibility for diverse chemical manipulations of the core structures. In total 29 compounds were selected (S1 Table) which showed low inter-replicate variability in primary and counter-screen with standard deviations (SD) < 30% (Fig 2C) (Fig 1, step 5).

**Table 1:** Cell type characteristics and proliferation potential.

Cell line	Cell type	Proliferation	Reference
**HOS**	OS	Low	ATCC
**Saos-2**	OS	Low	ATCC
**ZK-58**	OS	Low	ATCC
**MG-63**	OS	High	ATCC
**MNNG**	OS	High	ATCC
**U2OS**	OS	High	ATCC
**hFOB1.19**	hum OB ^a^	Low	[26,31]
**hOB**	hum OB ^b^	Low	Promocell
**L87/4**	hum MSC	High	[32,33]
**HEK293T**	emb kidney	High	ATCC
**HepG2**	HCC	Low	ATCC

The table shows cell type characteristics and proliferation potential of the different cell types used in this study. Abbreviations: OS, osteosarcoma; hum OB, human osteoblasts; hum MSC, human mesenchymal stromal cells; emb, embryonic; HCC, hepatocellular carcinoma.

^a^immortalized human osteoblasts

^b^primary human osteoblasts

Subsequent hit validation and analysis of dose-response relationship of the remaining 29 compounds in three OS cell lines (U2OS, HOS, Saos-2) and two non-OS control cell types (HepG2—hepatocellular carcinoma cell line, and hOB—primary human osteoblasts) finally identified two most prominent compounds, namely A13 and H12 with different structural characteristics (Fig 2D and 2E). The compound A13 had pyrazole and thiophene heterocyclic cores bridged by a benzamide moiety. The compound H12 contained a pharmacologically potent and biologically relevant chromene scaffold. These two amphiphilic compounds showed reasonable assay reproducibility with SD of 2% (Fig 2C).

**Fig 3:**
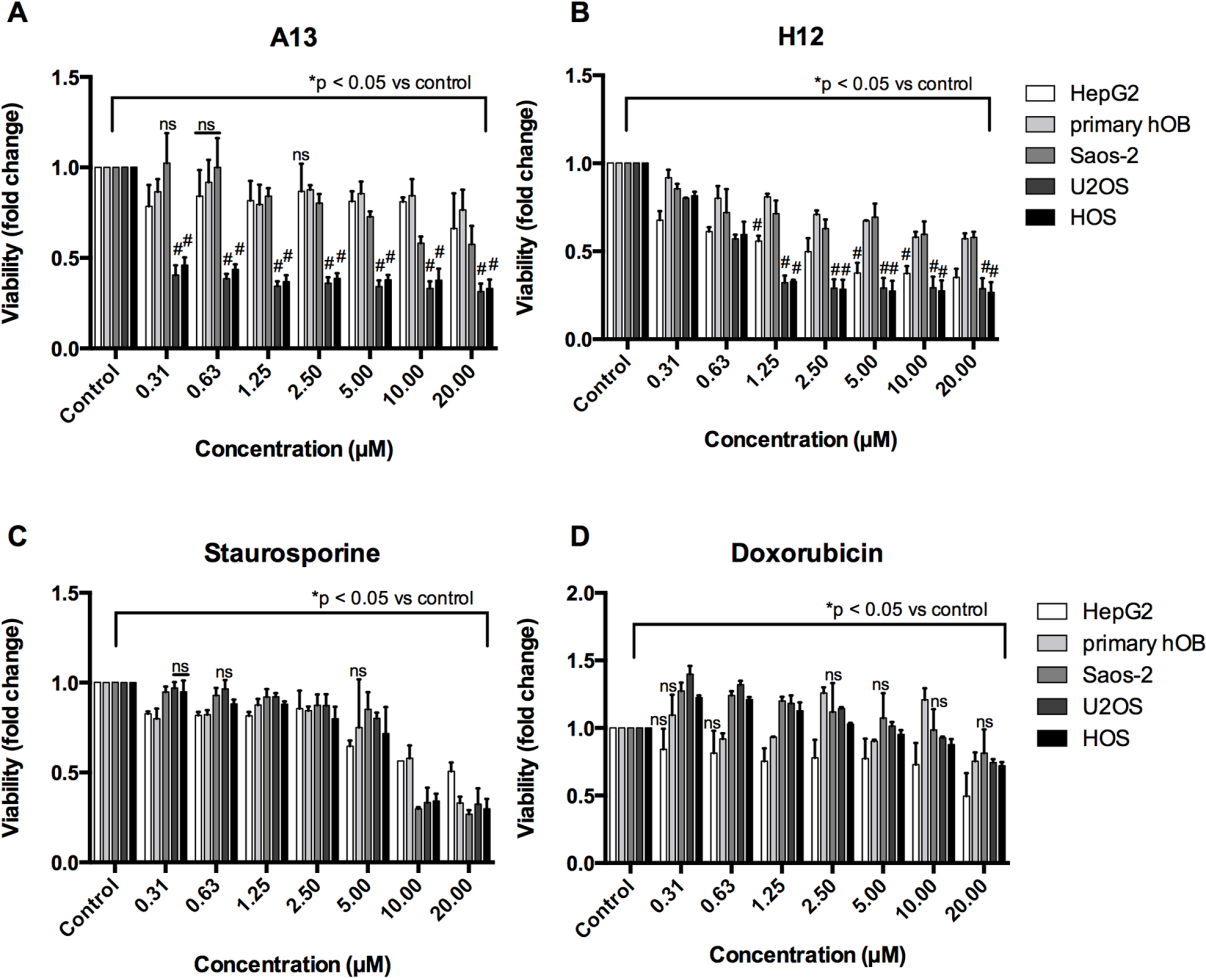
Dose-response analyses in OS cell lines and control cells after compound treatment. Cells were treated for 24h with compounds A13 (A), H12 (B), or with classical drugs staurosporine (C) and doxorubicin (D), respectively. Cell viability was assessed using CtB assay. Shown is fold change of cell viability normalized to DMSO-treated control for each cell type after incubation with increasing concentrations (μM) of the respective substance for 24h. Data are expressed as mean +/- SD from duplicates of three independent experiments. Differences of means were calculated using multiple t test for all cell lines versus primary hOB and all highly significant differences with #p < 0.001 are indicated by hash key (#). Differences of means to control were calculated for all bars and all were significantly different from control with *p < 0.05 indicated by the lines on top of the bars except those bars that are marked with ns = not significant (p > 0.05).

The compound A13 strongly reduced viability of OS cells (U2OS and HOS) at concentrations ranging between 0.31 and 20 μM (Fig 3A). In contrast, viability of HepG2 and primary hOB was not significantly affected. A strong selective effect (p < 0.001) in U2OS and HOS compared to primary hOB cells was observed at concentrations from 0.31 μM to 20 μM. Treatment with the compound H12 resulted in a significant dose-dependent decrease in cell viability of U2OS and HOS cells (Fig 3B). The compound H12 showed a significant selectivity (p < 0.001) in U2OS and HOS observed at concentrations between 1.25 μM and 20 μM when compared to primary hOB cells, but also in HepG2 (Fig 3B). Of note, in both treatments the p53-negative OS cell line Saos-2 showed almost no sensitivity. IC50 values for cell viability have been calculated for the compounds A13 and H12 (S1 Fig), and for staurosporine and are shown in.Table 2 The IC50 values for compound A13 and H12 clearly reflect the differences in sensitivity to compound treatments of the different cell types.

**Table 2:** IC50 values for the new compounds A13 and H12, and for staurosporine.

Cell line	A13 (μM)	H12 (μM)	Staurosporine (μM)
HepG2	108.70	2.3	n.d.
Primary hOB	44.54	n.d.	11.63
U2OS	0.21	0.89	6.69
HOS	0.28	0.81	6.00
Saos-2	48.50	n.d.	6.16

Dose-response curves for the indicated compounds and cell lines are shown in S1 Fig. In some cells and for doxorubicin in total the IC50 value could not be determined (n.d.), because no sigmoidal dose-response curves could be obtained.

The effectiveness of the new compounds was compared to two well-known drugs: Staurosporine, a pan-inhibitor of kinases, is a well-known research compound often used as a classical apoptosis-inducing agent [28], and doxorubicin that is used in clinical OS treatment and induces strong cytotoxicity [29]. Importantly, the U2OS cell line showed reduced sensitivity to this drug [30]. In our assay, staurosporine reduced the cell viability of all cell types at concentration of 20 μM or higher (Fig 3C). In contrast, doxorubicin only slightly affected cell viability at similar concentration ranges (Fig 3D). However, results showed that both the A13 and H12 compounds were effective in OS cell lines at concentrations lower that those observed for staurosporine and doxorubicin (≤1.25 μM vs. ≤ 20 μM, respectively). Taken together, the A13 and H12 compounds identified by our screening strategy had the most potent effects in U2OS and HOS cells

### Mechanistic aspects of compound action

To gain more insight into the mechanistic aspects of action of the two active compounds, a multiparametric assay was applied combining high-throughput imaging and quantification techniques. In this assay, several characteristic morphological changes are measured: cell loss, nuclear shrinkage, cell membrane disruption and mitochondrial dysfunction.

**Fig 4:**
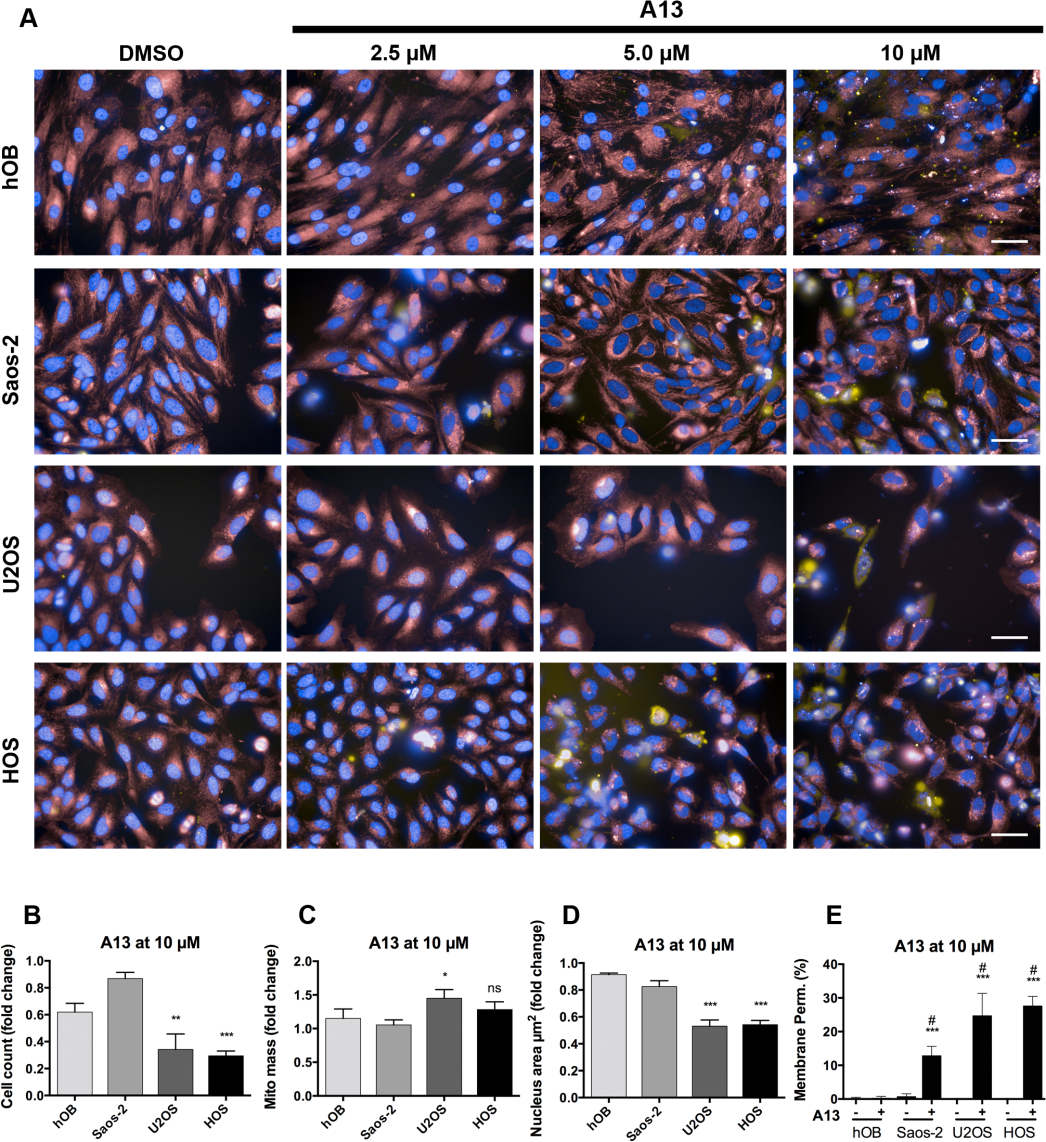
Multiparametric assay in OS cell lines and control cell types after compound A13 treatment. A) Immunofluorescence images show pseudo-colored nuclei (blue), mitochondria (red) and lysed cells (yellow). Cell type is indicated on the left, control (DMSO) and compound concentrations are indicated on the top of the images. Scale bars are 50 μM. Graphs in B)-E) show results from automated image quantification. The parameters cell count (B), mitochondrial (mito) mass (C), nucleus area (μm^2^) (D), and membrane permeability (%) (E) were determined for treatment with compound A13 at 10 μM for 24h. Unpaired t test showed *p < 0.05, **p < 0.01, ***p < 0.001, and not significant (ns) for U2OS vs. hOB and HOS vs. hOB, respectively, and in E) #p < 0.001 relative to DMSO-treated control of the respective cell type (A13^-^). Data are expressed as means +/- SD from duplicates of two independent experiments and are shown as percent or as fold change relative to DMSO-treated control.

The compound A13 induced in U2OS and HOS a loss in the number of cells, disruption of cell membrane, mitochondrial changes (increase of mitochondrial mass) and reduction in nuclear size at concentrations of 5 μM and 10 μM (Fig 4). Almost no dead cells and no changes in nucleus area were observed in Saos-2 and in the control cells hOB (Fig 4).

**Fig 5:**
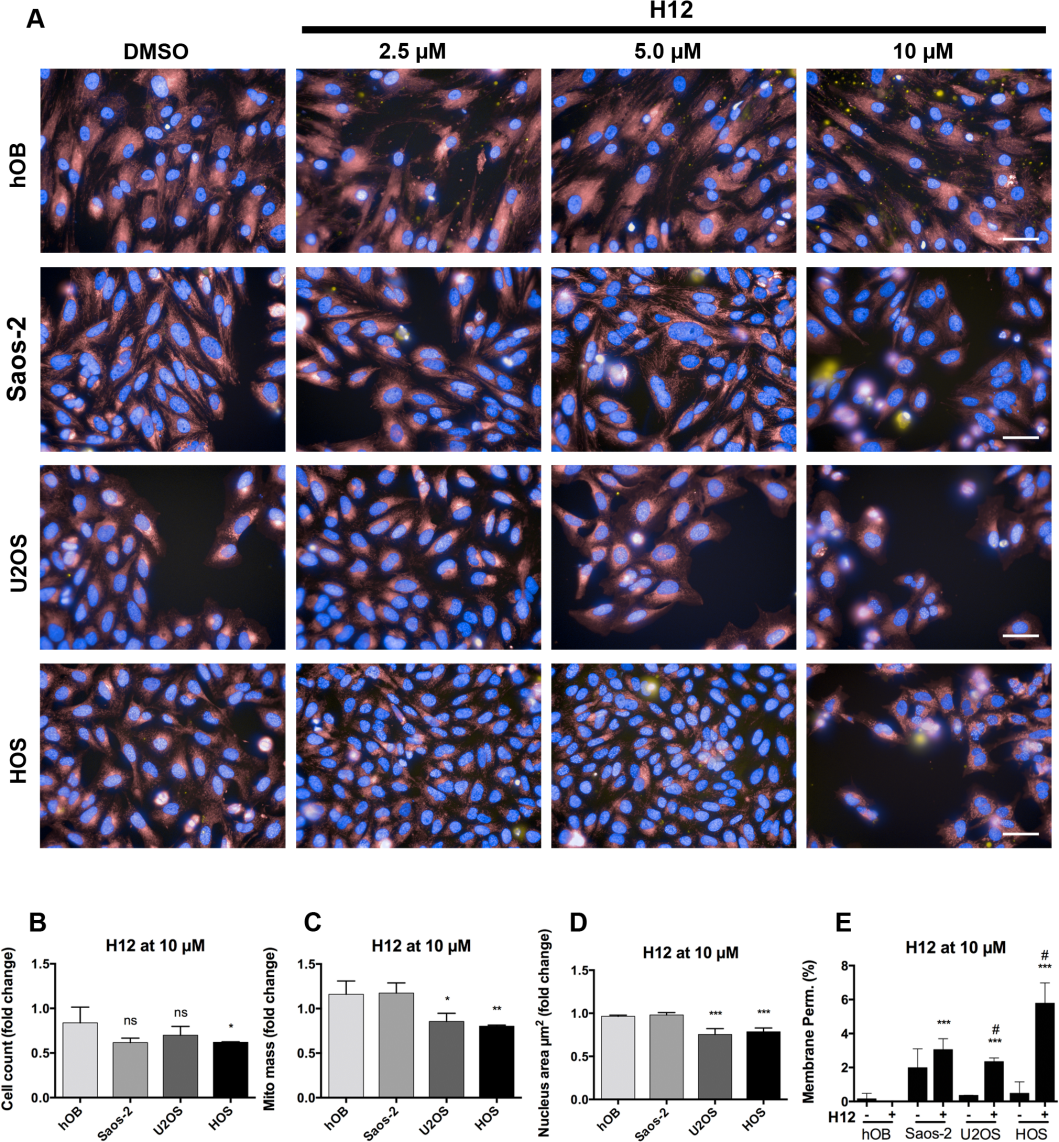
Multiparametric assay in OS cell lines and control cell types after compound H12 treatment. A) Immunofluorescence images show pseudo-colored nuclei (blue), mitochondria (red) and lysed cells (yellow). Cell type is indicated on the left, control (DMSO) and compound concentrations on the top of the images. Scale bars are 50 μM. B)-E) shows results from automated image quantification. The parameters cell count (B), mitochondrial (mito) mass (C), nucleus area (μm^2^) (D), and membrane permeability (%) (E) were analyzed after treatment with compound H12 at 10 μM for 24h. Unpaired t test showed *p < 0.05, **p < 0.01, ***p < 0.001, and not significant (ns) for U2OS vs. hOB and HOS vs. hOB, respectively, and in E) #p < 0.001 relative to DMSO-treated control of the respective cell type (H12^-^). Data are expressed as means +/- SD from duplicates of two independent experiments and are shown as percent or as fold change relative to DMSO-treated control.

Treatment with the compound H12 resulted in cell loss in U2OS and HOS, decreased mitochondrial mass and nucleus area and a slight increase in membrane permeability (Fig 5).

In general, these parameters indicate that the compounds A13 and H12 are capable to induce cell death in OS cell lines. Reduction of nuclear size most probably results from nuclear condensation indicating the induction of apoptosis by the two compounds. However, treatment with compound H12 showed reduced effects in OS cell lines compared to the compound A13.

### The apoptosis signaling in OS cells after compound treatment

**Fig 6:**
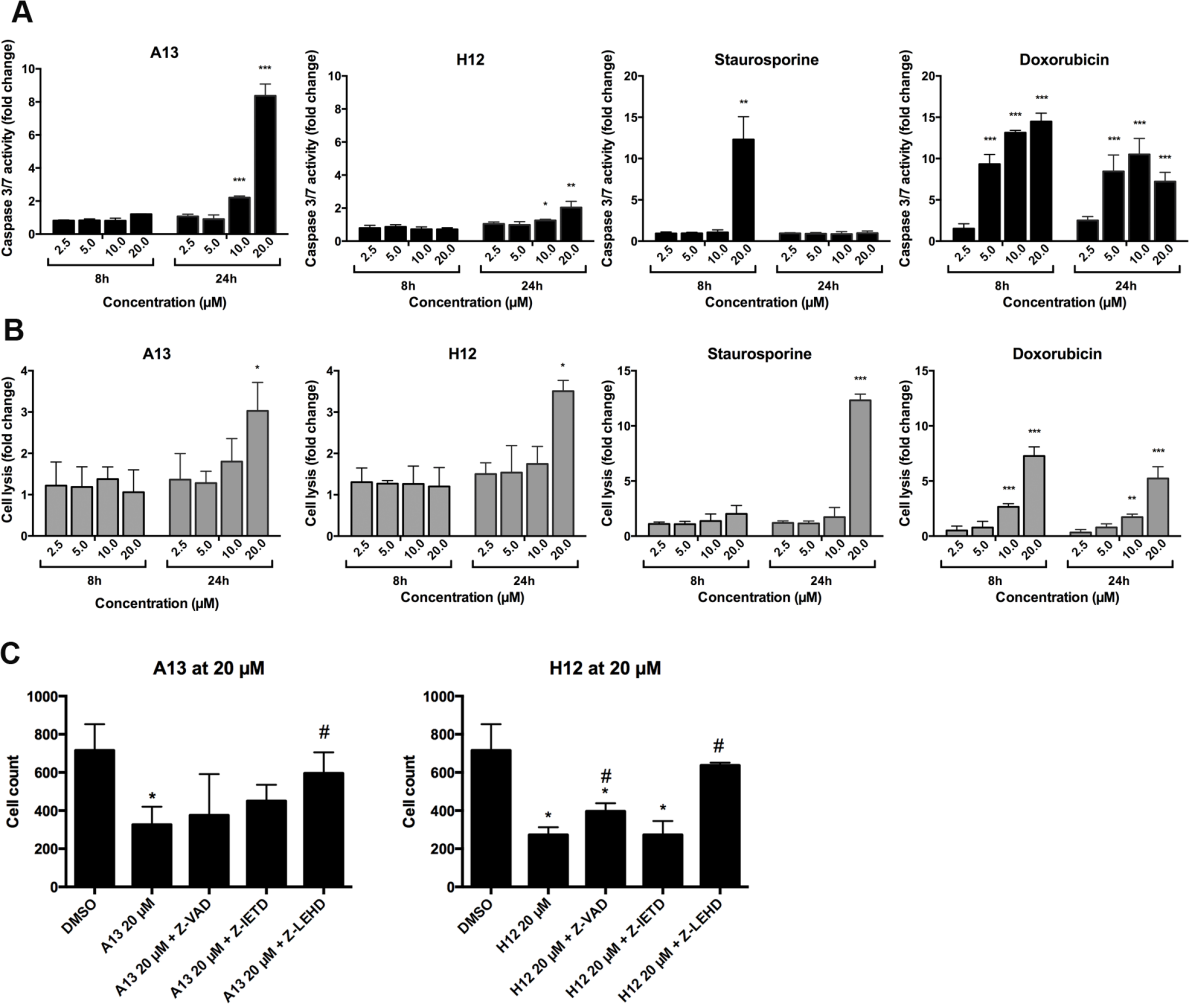
Induction of caspase 3 and 7 activity and cell lysis in U2OS cell line after compound treatment. A) shows caspase 3/7 activity and B) cell lysis for U2OS cell line treated with increasing concentrations of compound A13, H12, staurosporine and doxorubicin, respectively. Caspase 3/7 activity was determined after 8 and 24h of treatment using Caspase-Glo 3/7 Assay. Cell lysis was determined using CellTox Green Cytotoxicity Assay. Data are shown as fold change of mean +/- SD from duplicates of two independent experiments relative to DMSO-treated control. Unpaired t test was performed versus DMSO-treated control with *p < 0.05, **p < 0.01, and ***p < 0.001. C) shows cell count determined by quantification of Hoechst-stained nuclei after treatment of HOS cells with 20 μM of compound A13 and H12, respectively, and after co-treatment of cells with compounds and caspase inhibitors (pan-caspase inhibitor Z-VAD-FMK, caspase 8 inhibitor Z-IETD-FMK or caspase 9 inhibitor Z-LEHD-FMK) for 24h. Means and SD from duplicates of one representative experiment of two are shown (*p < 0.05 versus DMSO treated control; #p < 0.05 versus cells treated with 20 μM of compound).

To analyze in more detail pathways involved in cell death mechanism, we determined caspase 3/7 activity and cell lysis in U2OS cells. Cells were treated for 8 and 24h with compounds and caspase 3/7 activity was determined and compared to the activity of cells treated with staurosporine and doxorubicin (Fig 6). Caspase 3/7 activation was strongly increased after treatment with compound A13 at a concentration of 10 and 20 μM, while H12 only slightly induced caspase activity at a concentration of 20 μM for 24h (Fig 6A). Both compounds also led to a moderate induction of caspase 3/7 activity in primary hOB (2-fold) and Saos-2 (3 to 4-fold) after 24h (S2 Fig). In comparison, staurosporine strongly induced caspase 3/7 already after 8h, and doxorubicin most effectively induced caspase 3/7 activity after 8 and 24h treatment (Fig 6A). Despite strong caspase activation by compound A13, the observed cell lysis was rather low (up to 3-fold at the concentration of 20 μM after 24h) and was similar to that observed for compound H12 (Fig 6B). In cells treated with staurosporine, however, classical apoptosis induction was observed by initial caspase activation followed by time delayed strong cell lysis (Fig 6A and 6B). Doxorubicin showed dose-dependent increase of cell lysis at both time points (Fig 6B). Taken together, both compounds were capable to induce caspase 3/7 activity.

To test whether the compound effects are dependent on caspase activity we investigated the effects of several caspase inhibitors (pan-caspase-, caspase 8-, and caspase 9 inhibitors) in co-treatment with compound A13 and H12. We observed a significant decrease of cell number in cells treated with 20 μM of A13 and H12, respectively (Fig 6C). These effects were significantly reduced in the presence of the caspase 9 inhibitor Z-LEHD-FMK. The pan-caspase inhibitor Z-VAD-FMK also reversed the effects of compound H12 in a significant manner while the caspase 8 inhibitor Z-IETD-FMK had no effect (Fig 6C). The results indicate an action of the compounds on caspase 9 and the involvement of intrinsic apoptosis pathways in the drug responses.

## Discussion

In this study, we demonstrate two new compounds identified by our screening strategy as potential candidates for OS treatment. The two compounds, namely A13 and H12, had strong and dose-dependent effects on cell viability in the OS cell lines U2OS and HOS, but did neither significantly affect the control cell lines from hepatocellular carcinoma (HepG2) nor primary human osteoblasts. The compounds induced changes in cell morphology that included nuclear condensation and changes in the mitochondrial mass. In addition we observed a strong (A13) and a moderate (H12) induction of caspase 3/7 activation in U2OS cells. These results indicated the potential of apoptosis induction in OS cells by the two new compounds.

Induction of apoptosis is an important issue in therapy development not lastly because this cell death mechanism prevents an inflammatory response that is the case in necrosis [34]. Consequently, the potential of a compound to induce necrosis should be limited to the minimum since inflammation induced by cells dying from necrosis causes severe side effects in patients. Morphological indicators of apoptosis are amongst others nuclear condensation and intact cell membrane whereas hallmarks of necrotic cells are cell lysis and mitochondrial swelling [35]. *In vitro* the late stages of apoptosis also include cell lysis [36]. In our study, a strong cell lysis subsequent to caspase activation was observed for the well-known apoptosis inducing agent staurosporine. Our data showed that the compounds did not induce strong necrosis in OS cells at the concentrations investigated since the cell lysis observed was rather low. Moreover, the morphological changes that we observed in OS cells are associated with apoptosis at least at the doses investigated.

The results obtained from the caspase 3/7 activity assay suggested the time point of apoptosis induction to be around 24h after compound treatment at concentrations of 10 to 20 μM. Using western immunoblotting, however, we could not detect cleavage of the apoptosis marker Poly (ADP-ribose) polymerase (PARP) at 10 μM after 24h, neither after 48 nor 72h (data not shown). In contrast, higher compound doses (30 μM) induced a strong PARP cleavage in HOS and Saos-2 cells and also in osteoblasts (hFOB1.19) (S3 Fig). Almost no protein was detected in U2OS cells, which may result from strong cell lysis at this high compound doses in the sensitive U2OS cell line [37]. We conclude that the cell line-selective effects that were strongly suggested using the cell viability assay (Fig 3, S1 Fig) and IC50 values (Table 2) are apparent at low compound concentrations (< 10 μM), while at higher doses the effects also affect other cell lines. In addition we performed Annexin V staining in combination with the dye Yo-Pro that stains lysed cells. We only observed Annexin V positive cells after A13 and H12 treatment between 5 to 10% Annexin V positive/ Yo-Pro negative cells in U2OS, HOS and Saos-2, whereas Annexin V positive cells were not induced after treatment in osteoblasts and HepG2 (S4 Fig). We conclude that the additional experiments support our findings that the compounds are capable of apoptosis induction, however the effects on apoptosis induction are rather mild which may argue for specificity of the effect without inducing general toxicity. More important, at lower doses the two compounds were almost not toxic to cells derived from hepatocellular carcinoma (HepG2), a cell line often used in drug discovery to exclude hepatocytotoxicity [38], and human osteoblasts.

The two compounds strongly reduced cell viability, whereas the induction of caspase 3/7 and cell lysis by the compounds were rather low. In contrast, staurosporine and doxorubicin strongly induced caspase 3/7 and cell lysis but effects on cell viability were only observed at higher concentrations (Fig 3). We assume that the strong induction of caspase 3/7 and cell lysis by doxorubicin (Fig 6) could be a bias of the fluorometric/luminogenic assays which leads to an overestimation of the signal, as has already been reported [39]. Our data, however, indicate that the cell viability assay, which measures the metabolic activity of a cell, implicates a strong reduction of cell viability in OS cells already at low concentrations. In contrast, an apparent induction of cell death was not detectable at low compound concentrations in the other assays. The discrepancy between the strong effects on cell viability (Fig 3A and 3B) and the modest effects on apoptosis induction (Fig 6A and 6B) and loss in cell number (Fig 5B) is explainable by the time course of the process. The cell viability reflects the metabolic activity of a cell that is not necessarily directly linked to apoptosis as an outcome. The effects on viability are present already at early time points and at lower compound concentrations, while apoptosis induction or other cell death mechanisms that finally lead to a detectable loss in cell number occur subsequent to that initial event [40]. Moreover, in Fig 6C we used increased concentrations of compounds A13 and H12 (20 μM) that led to a significant loss in cell number. We conclude that, beside a possible overestimation of doxorubicin effects, the two new molecules were capable to affect OS cells already at lower concentrations when compared to the well-established drugs.

The multiparametric assay also indicated that the A13 compound led to the increase of mitochondrial mass. In contrast, the H12 compound decreased the mitochondrial mass. Interestingly, the effects of the two compounds on cell number were significantly abrogated in the presence of the caspase 9 inhibitor Z-LEHD-FMK (Fig 6C), and also partly by the pan-caspase inhibitor Z-VAD-FMK, which indicates the involvement of intrinsic apoptosis pathways in the drug responses of the two compounds. For detailed analysis of apoptosis mechanisms the compound action on mitochondria will be further investigated in future studies. This is of particular interest because mitochondria are important players in mediating cell death and apoptosis and promising target structures in cancer therapy [41]. For example, analysis of mitochondria isolated from treated cells could give more insights into mitochondrial conditions [38]. Recently, Ma and colleagues described a pancratistatin analogue JC-TH acetate-4 (JCTH-4) and showed that this agent induced apoptosis in OS cell lines via mitochondrial targeting. Addition of the natural herb curcumin, that has anti-proliferative properties, further enhanced the apoptotic effects [42]. Moreover, they showed that JCTH-4 also induced autophagy in OS cells, a mechanism that has distinct outcomes in therapeutic usage and also depends on the p53 status of a cell [43]. Further downstream, autophagy may influence the PI3K/AKT/mTOR pathway that is also implicated in OS [44,45] thus making JCTH-4 a promising agent, although the effect of autophagy in cancers yet is not well characterized [34]. Thus it would be interesting to test whether OS cells treated with the two new molecules described in our study would also induce changes associated with autophagy and whether this would involve the PI3K signaling pathway.

With regard to the important role of p53 in many cancers and especially in OS the p53-negative OS cell line Saos-2 exhibited lower (compound A13) or almost no sensitivity (compound H12) to the newly identified compounds. This makes p53 and its associated pathways an interesting starting point for further investigations on the compound actions. It has to be noted that the OS cell lines used in this study are of different p53 status [25] and in some cell lines p53 expression has been associated to MDM2 (murine double minute-2) gene amplifications [25,46]. Moreover, differentially activated forms of p53 protein in Saos-2, U2OS and MNNG-HOS cells have been implicated in drug sensitization of OS cells [47] which makes the role of p53 very complex and difficult to elucidate. A greater sensitivity of p53-wild type cells towards PI3 kinase inhibitor has been already reported in glioma cells [48]. PI3 kinases that act downstream of p53 in the PI3K/AKT/mTOR pathway are implicated in OS thus being interesting targets of small molecule inhibitors [2,9,49]. In future studies we will investigate the important link between p53 and the sensitivity of OS cell lines to the two new compounds identified in this study. Here, we aim to test these two compounds after the knockdown of p53 in OS cells or after the re-introduction of an active p53 into Saos-2 to further elucidate the role of p53 [48].

Considering the severe side effects induced by the multidrug chemotherapy applied in OS, new molecules exhibiting such selective effects as described in our study are of special interest in drug development. In recent years, many molecules discovered for therapeutic application have evolved from phenotypic screens by identification of compounds exhibiting a certain action of interest, such as an anti-proliferative or an apoptosis inducing effect [50]. Thus, the compounds identified in our study could crucially contribute to the development of new drugs in OS.

## Summary

The discovery of new drugs and the improvement of treatment strategies still is an important issue in cancer disease because such new drugs could support or even substitute a conventional therapy when its effectiveness is compromised. In this study, we used a phenotypic high-throughput approach to screen the effectiveness of a 25,000 small-molecule diversity library against osteosarcoma cells. We characterized a large set of diverse compounds in U2OS cell lines and selected compounds that actively reduced the cell viability. By applying various OS and non-OS cells, we then step-by step could exclude inactive or overall toxic compounds and tested the remaining compounds for their chemical properties and robust dose-response. Finally, we identified two new small molecules that selectively reduced viability and induced apoptosis signaling in OS cell lines in a dose-dependent manner. The two compounds were robust in medicinal chemistry analysis and are susceptible to chemical manipulations due to their structural properties. Thus, we provide promising substances affecting OS cell viability that offer a possibility for further drug development in OS.

## Materials and Methods

### Library buildup and compound preparation

The composition of the 25,000 diverse-compound library has been described earlier [19]. Compounds were either dissolved in dimethylsulfoxide (DMSO) arranged in 384-well micro-plates for high-throughput screening (HTS) or delivered as a dry powder in glass vials for individual assays. All compounds were diluted in DMSO (product number AE56.2; Roth, Karlsruhe, Germany) and stored tightly sealed at -20°C. The two compounds found in the screening were obtained from ChemDiv (San Diego, CA), and can be re-ordered using the compound IDs for A13 (compound ID: 6228–2300) and H12 (compound ID: 5948–4347), respectively. Staurosporine (s5921) and Doxorubicin (d1515) were both obtained from Sigma-Aldrich (Taufkirchen, Germany) and diluted in DMSO and H_2_O dest., respectively.

### Screening instruments and performance

Cell seeding and assays were performed in black 384-well microplates (Greiner Bio-One, Monroe, NC; 781091) for fluorometric assays, and white 384-well Optiplate (PerkinElmer—PE, Waltham, MA; 6007290) for luminescence-based assays, respectively. Cells were seeded 24h before treatment in 384-well microplates with a cell number of 2000 cells/well for highly proliferating cells and 3000 cells/well for low proliferating cells (**Table 1**) [51]. This resulted in an intermediate confluency after 24h (time of compound addition) and in a confluent layer after approximately 48h. Plate handling, automated cell seeding and compound addition was performed using following HTS devices: Plate handling was performed using a Sciclone G3 with a Twister II robotic arm (PerkinElmer, USA) and a Flexdrop (PerkinElmer, USA) liquid handling system. CellTiter Blue (G8081; Promega, Mannheim Germany) measurements were performed using an EnVision Multilabel Reader (PerkinElmer, USA).

### Cell lines and primary cells

Cell lines (U2OS, HOS, Saos-2, ZK-58, MNNG-HOS, MG-63, hFOB1.19) were obtained from American Type Culture Collection (ATCC) (Manassas, VA) or from partner institutes (HepG2, HEK293T, L87/4) (see Table 1 for details). Characteristics of OS cell lines are described in [25,52]. Authentication of OS cell lines was done by Eurofins GmbH and confirmed by online STR-matching analysis (www.dsmz.de/fp/cgi-bin/str.html). All cells despite Saos-2 cells were grown in RPMI 1640 (Invitrogen, Karlsruhe, Germany) with L-Glutamine, 10% fetal calf serum (FCS), 100 U/ml penicillin, and 50 μg of streptomycin. Saos-2 cells were grown in the same medium containing 15% FCS. Primary human osteoblasts (hOB) were obtained from Promocell (C12720; Heidelberg, Germany) and cultured in osteoblast growth medium containing 10% supplement mix (Promocell; C27001). For assay performance, medium of Saos-2 and hOB cells was replaced by RPMI 1640 with L-Glutamine, 10% FCS, 100 U/ml penicillin, and 50 μg streptomycin at time of compound addition. Primary cells were used for experiments in passages three to five.

### Fluorometric cell viability assay

Cell viability was determined using Celltiter Blue (G8081; Promega, Mannheim Germany). This assay measures the reducing potential according to metabolic activity in cells. Cells were seeded 24h before treatment in black 384-well plates. Following 24h incubation at 5% CO_2_, 37°C, compounds (10 mM) were added to the cells in a final concentration of 10 μM (0.1%. Cells were incubated for further 22h. Pre-warmed Celltiter Blue reagent was added to the medium (1:5 dilution), plates were gently shaked and incubated for 2h at 5% CO_2_, 37°C. Prior to measurement, plates were again gently shaked and cell viability was measured at the Envision Multilabel Reader (PE) at 560_Exc_/590_Em_ nm (4 areas per well measurement). Data were normalized to vehicle control-treated cells (1% DMSO).

### Fluorometric cytotoxicity assay

The CellTox Green Express Cytotoxicity Assay (G8731; Promega) contains a fluorescent dye that binds to DNA of cells with impaired membrane integrity (cell lysis). Binding of the dye to cell DNA results in fluorescence signal which is detectable at 495 nm. For determination of cell lysis, cells were seeded manually in black 384-well microplates and incubated 24h at 5% CO_2_, 37°C. Medium was replaced by fresh medium containing the fluorescent dye diluted by 1:500 dilution. Compounds and control substances were diluted in the respective medium/dye mix and added to the cells. For positive control of cell lysis, 30 μM digitonin was added to the cells 15 min prior to measurement. Background signal (medium and CellTox Green dye) was subtracted and data were normalized to vehicle control (1% DMSO).

### Luminescent caspase 3/7 activity assay

The caspase 3/7 assay (G8091, Promega) uses a DEVD-linked luminogenic substrate that is cleaved upon exposure to active caspase 3 and 7. Cleavage of the substrate results in a luminescent signal generated by activated luciferase. For apoptosis induction, cells were seeded in white 384-well microplates and incubated for 24h at 5% CO_2_, 37°C. Subsequently, compounds and control substances were added to the cells in a dilution series and incubated for further 8 and 24 h, respectively. One hour before end of drug exposure time, Caspase- 3/7-Glo reagent was added to the cells in 1:2 dilution as described by the manufacturer and incubated for one hour at room temperature in dark. Luminescence was measured at 700 nm. Background signal (medium and Caspase- 3/7-Glo reagent only) was subtracted and data were normalized to DMSO-treated cells.

### Multiparametric cytotoxictity assay

The multiparametric assay determines cell number, cell membrane integrity and nuclear fragmentation in an image-based quantification using the Operetta/Harmony high-throughput imaging platform (PE) [38]. Cells were seeded into black 384-well micro-plates 24h prior to treatment. For co-treatment of cells with compounds and caspase inhibitors the cells were pre-treated with pan-caspase (Z-VAD-FMK), caspase 8 (Z-IETD-FMK) or caspase 9 inhibitor (Z-LEHD-FMK; all obtained from BioVision Incorp., CA) in a 1:1000 dilution 30 min prior to compound addition. Compounds were added in a serial dilution to the cells and plates were incubated for 24h at 5% CO_2_, 37°C. For DNA counterstaining, NucBlue, a dye similar to Hoechst 33342, was used, mitochondria were stained using MitoTracker deep red and dead cells were stained using Po-Pro iodide (product numbers: R37605, M22426, and P3585, respectively; all by Life Technologies). Dyes were diluted in medium and added to living cells. After 45 min incubation at 5% CO_2_, 37°C, images were recorded using the automated Operetta microscope using the 40x NA objective for high-resolution images. For quantification, two images of each condition in duplicates and for each cell type were recorded using a 20x objective. This resulted in a cell number of approximately 500 cells of each condition. Quantification on cell size was performed using the Harmony software (PE).

### Statistical analysis

All the data were collected in the given precision in tab-separated text formats and Microsoft Excel. Hierarchical clustering of cell viability data was performed using the R software environment for statistical computing and graphics version 3.0.1 (www.r-project.org, package 'cluster', function: agnes, agglomerative hierarchical clustering, metric: Pearson correlation, method: complete linkage). In order to determine differences of means the t-test (unpaired, two sample test, p values less than 0.05 were considered to be significant) was applied using GraphPad Prism (GraphPad Software 6.0f, La Jolla California, USA, www.graphpad.com). In the case of multiple tests we corrected the raw probability values by the FDR method [53]. In the drug discovery process IC50 values (inhibitory concentrations) were calculated to evaluate the suitability and performance of a drug [54]. The calculation of the IC50 values were performed with GraphPad Prism and followed a nonlinear regression model applied to the sigmoidal dose-response curves of the cell viability data. The values were log-transformed before fitting the model. Details to the procedure can be found in the Graphpad Prism User Guide (www.graphpad.com/guides/prism/6/user-guide).

## Supporting Information

S1 Fig Dose-response curves of cell viability in the five cell lines used for hit validation (HepG2, primary hOB, U2OS, HOS, Saos-2) shown for the compound A13 and H12. Curves were generated from nonlinear regression and IC50 values have been calculated. Cell viability has been determined using the Celltiter Blue Assay after compound incubation for 24h. Data represent means and SD of two (HepG2, hOB) to three (U2OS, HOS, Saos-2) independent experiments always performed in duplicates.
(TIFF)

S2 Fig Caspase 3/7 activity and cell lysis assays for primary hOB and Saos-2 cells 24h after compound treatment. Primary hOB and Saos-2 cells were treated with 10 or 20 μM of compound A13 or H12 or with vehicle-control (DMSO). Slight to moderate induction of caspase 3/7 activation was observed for the two cell types (2-fold for hOB, 3 to 4-fold for Saos-2). Cell lysis was significantly increased in hOB (2- to 3-fold). Bars show means and SD of duplicates of one representative experiment of two. Fc = fold change relative to DMSO-treated cells, *p < 0.05, **p < 0.01, ns = not significant p > 0.05.
(TIFF)

S3 Fig Western immunoblotting of Poly (ADP-ribose) polymerase—PARP and cleaved PARP as an indicator of apoptosis in whole cell protein extracts from cells treated with high doses (30 μM) of compound A13 or H12 for 24h. Increase of cleaved PARP and in parallel decrease of full-length PARP was observed in HOS, Saos-2 and hFOB1.19 for both compounds. In U2OS, almost no protein could be detected which may be a result of the high dose treatment leading to strong either primary or secondary necrosis in which proteins have already been degraded.
(TIF)

S4 Fig Analysis of phopsphatidylserine externalization on cell membranes by Annexin V/ Yo-Pro staining and flow cytometry. Cell were incubated with 30 μM of compounds (A13 or H12) or with vehicle control (DMSO) for 20h and stained for Annexin V. Yo-Pro was used to determine lysed/necrotic cell fraction. Yo-Pro negative cells were excluded from the final analysis by gating to Yo-Pro negative/ Annexin V positive cells. Images show Annexin V versus forward scatter (FSC) with gating on Annexin V positive cells (R5). % of Annexin V positive cells are indicated.
(TIFF)

S1 Table Cell viability of various cell lines from primary and counter screen treated with the 29 short-listed compounds. Viability (%) is shown for the indicated cell lines after treatment with the compounds named after their hit-picking well-ID. The table also shows the chemical formula and the molecular weight for each of the 29 compounds.
(XLSX)

S1 Text Additional materials and methods. (DOCX)
