# Supplementary figures and images for: New Small Molecules Targeting Apoptosis and Cell Viability in Osteosarcoma
Source: PLoS One. 2015 Jun 3;10(6):e0129058. doi: 10.1371/journal.pone.0129058 (PMC4454490; doi:10.1371/journal.pone.0129058)

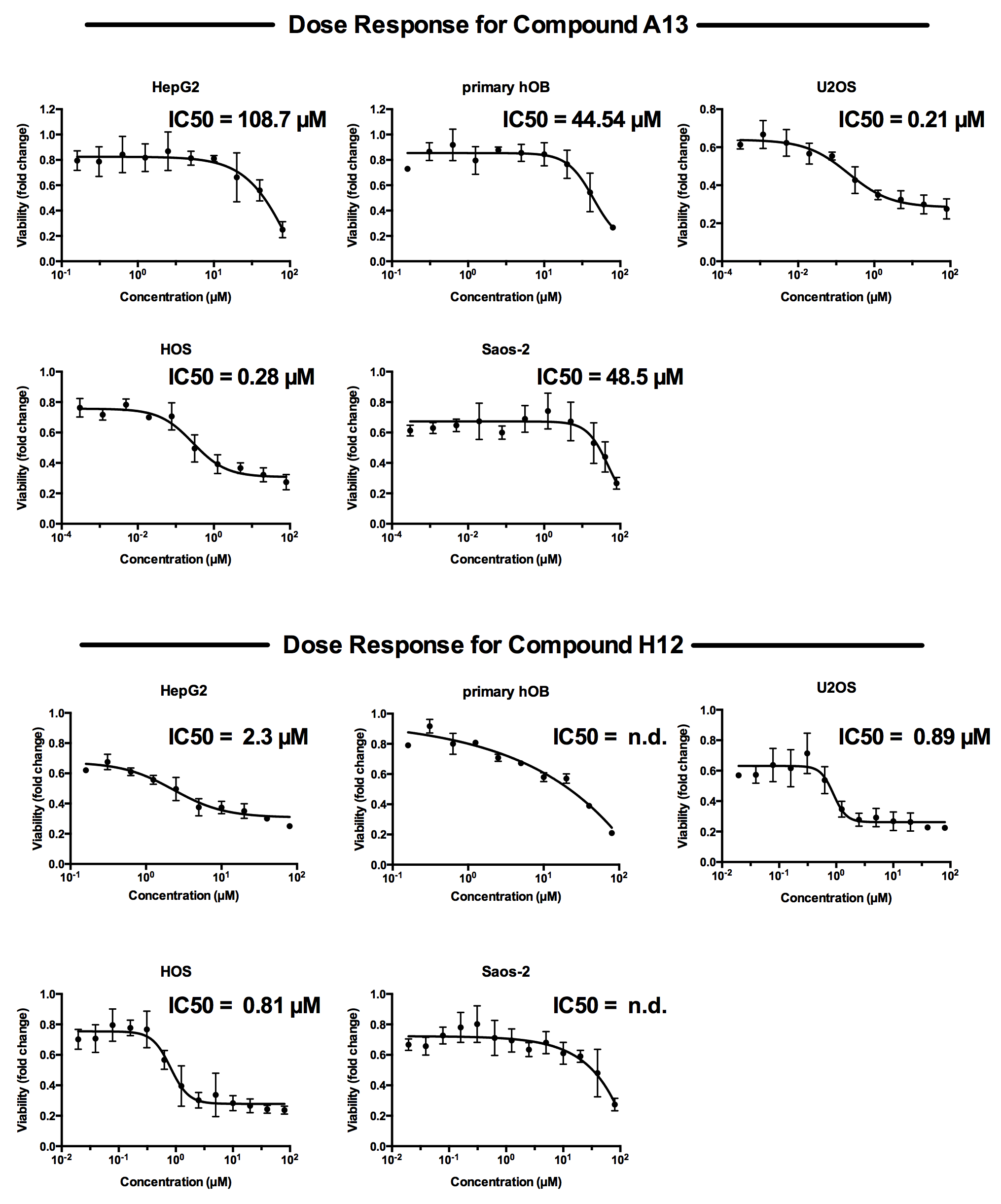

Supplement: S1 Fig — Curves were generated from nonlinear regression and IC50 values have been calculated. Cell viability has been determined using the Celltiter Blue Assay after compound incubation for 24h. Data represent means and SD of two (HepG2, hOB) to three (U2OS, HOS, Saos-2) independent experiments always performed in duplicates. (TIFF) [file pone.0129058.s001.tiff]

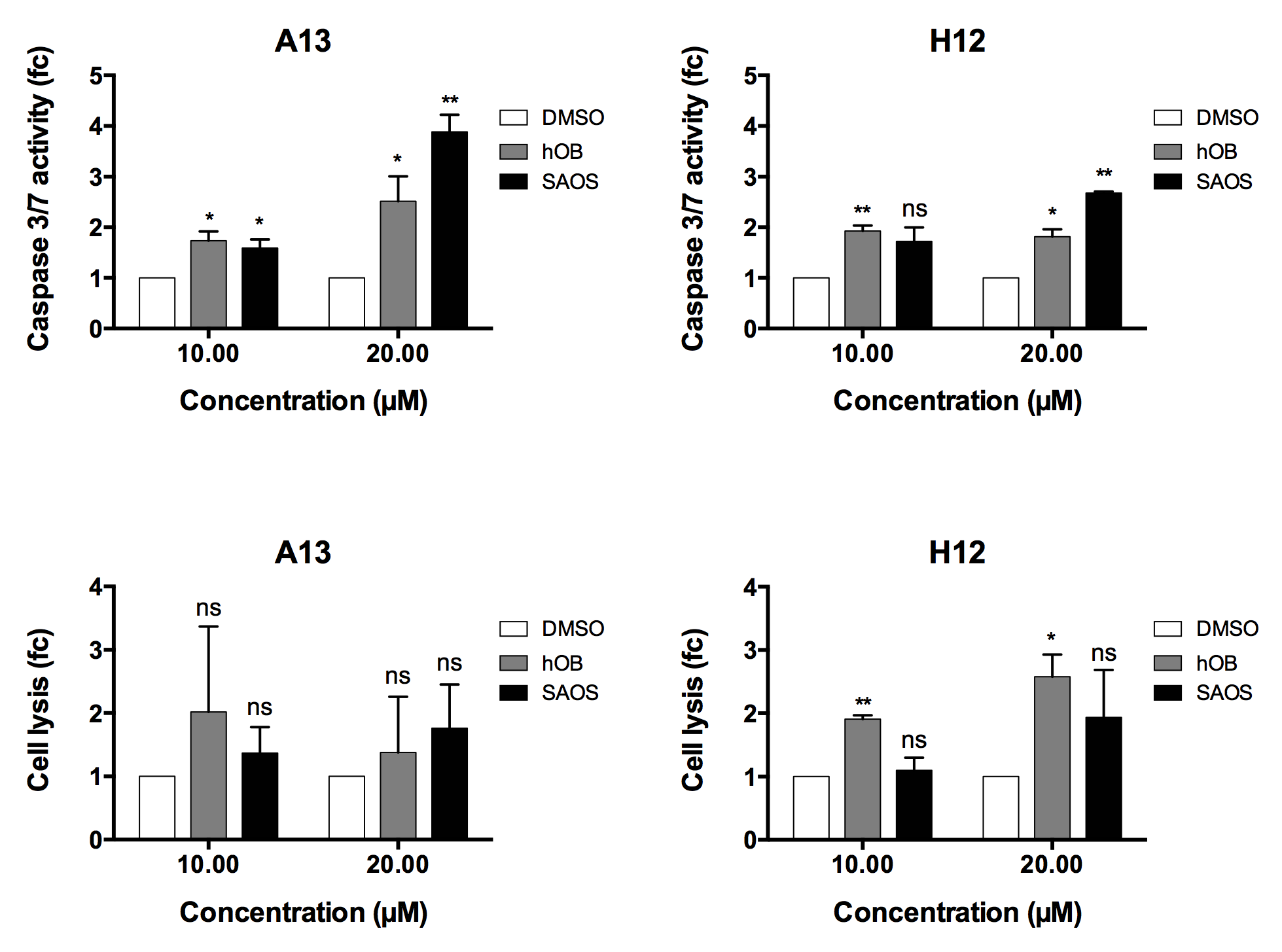

Supplement: S2 Fig — Primary hOB and Saos-2 cells were treated with 10 or 20 μM of compound A13 or H12 or with vehicle-control (DMSO). Slight to moderate induction of caspase 3/7 activation was observed for the two cell types (2-fold for hOB, 3 to 4-fold for Saos-2). Cell lysis was significantly increased in hOB (2- to 3-fold). Bars show means and SD of duplicates of one representative experiment of two. Fc = fold change relative to DMSO-treated cells, *p < 0.05, **p < 0.01, ns = not significant p > 0.05. (TIFF) [file pone.0129058.s002.tiff]

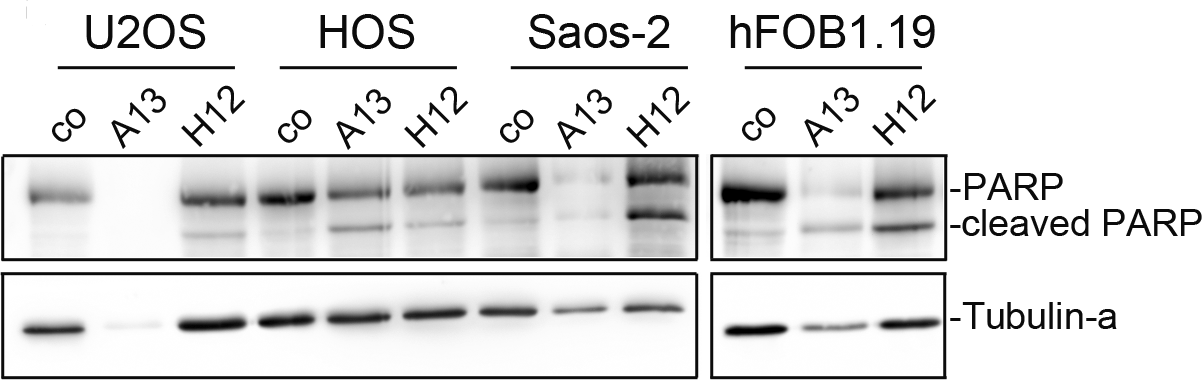

Supplement: S3 Fig — Increase of cleaved PARP and in parallel decrease of full-length PARP was observed in HOS, Saos-2 and hFOB1.19 for both compounds. In U2OS, almost no protein could be detected which may be a result of the high dose treatment leading to strong either primary or secondary necrosis in which proteins have already been degraded. (TIF) [file pone.0129058.s003.tif]

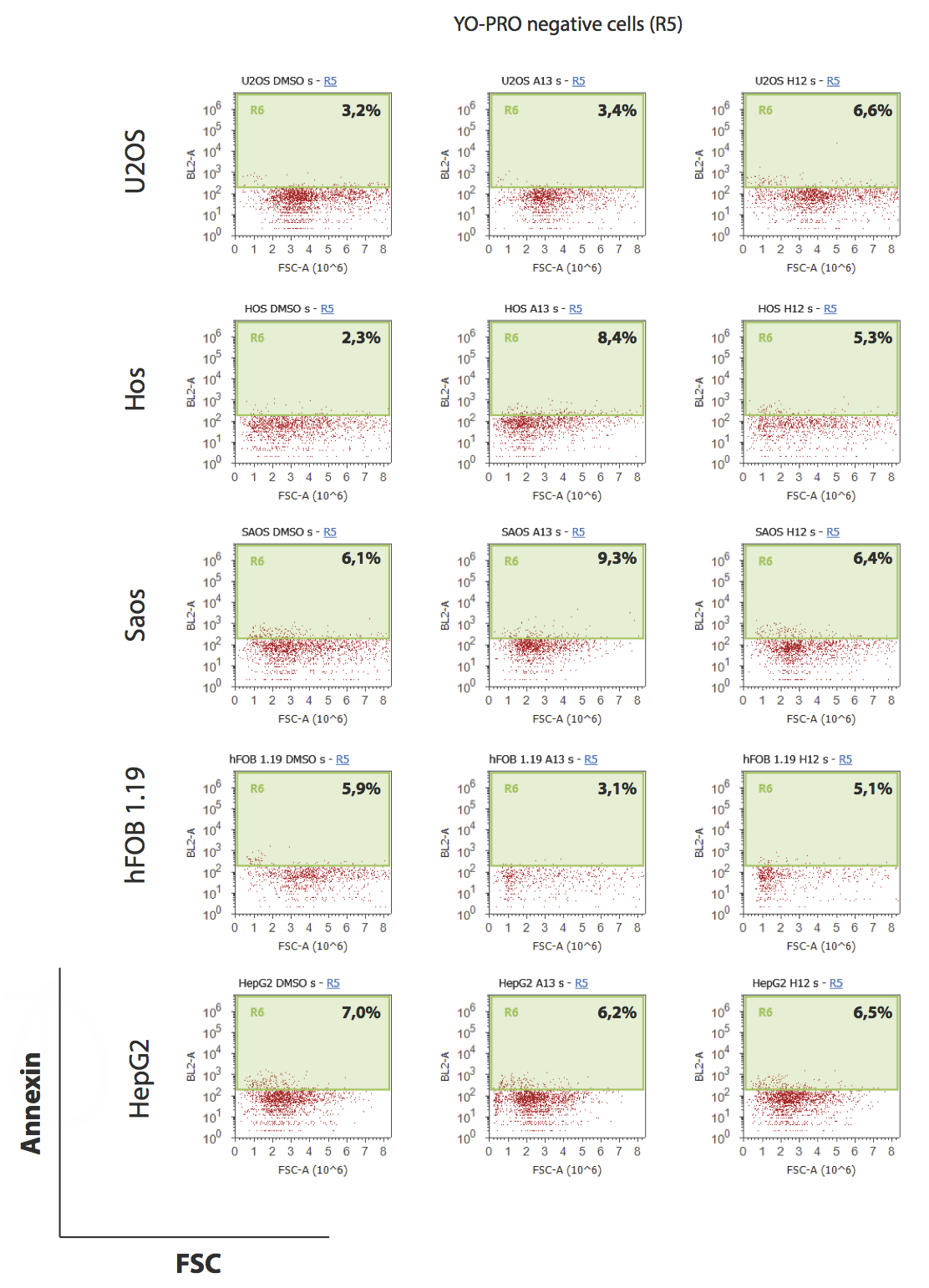

Supplement: S4 Fig — Cell were incubated with 30 μM of compounds (A13 or H12) or with vehicle control (DMSO) for 20h and stained for Annexin V. Yo-Pro was used to determine lysed/necrotic cell fraction. Yo-Pro negative cells were excluded from the final analysis by gating to Yo-Pro negative/ Annexin V positive cells. Images show Annexin V versus forward scatter (FSC) with gating on Annexin V positive cells (R5). % of Annexin V positive cells are indicated. (TIFF) [file pone.0129058.s004.tiff]
