# Supplementary material for: New Small Molecules Targeting Apoptosis and Cell Viability in Osteosarcoma
Source: PLoS One. 2015 Jun 3;10(6):e0129058. doi: 10.1371/journal.pone.0129058 (PMC4454490; doi:10.1371/journal.pone.0129058)
Supplement: S1 Text — (DOCX) [file pone.0129058.s006.docx]

# Supplementary Information Text

## Supplementary Material and Methods

### Western Immunoblotting

Whole cell proteins were extracted using lysis buffer containing 120 mM NaCl, 25 mM Tris-HCl pH 7.5, Triton X-100 1%, Phenylmethanesulfonylfluoride (PMSF) 1 mM, Naorthovanadat 1 mM. Pellets were resuspended in 100 µl of lysis buffer and incubated for 60 min on ice. Following a centrifugation step at 16,000 g, 4°C for 10 min the supernatant was transferred to a fresh tube. BCA assay (Pierce BCA Protein Assay Kit, Life Technologies) was used to determine protein concentrations according to manufacturer’s instruction. Protein solution was diluted accordingly in Laemmli buffer and 15 µg of proteins were loaded onto a 10 % SDS polyacrylamide gel. Western immunoblotting was performed as previously described (1). The following antibodies were used for immunodetection: anti-rabbit PARP and cleaved PARP (46D11) (#9532) and anti-mouse Tubulin-alpha (DM1A) (#3873) by Cell Signaling technologies.

### Annexin V/ Yo-Pro staining and flow cytometry

Cells were seeded in 6-well microplates and incubated for 24h at 5% CO_2_, 37°C. Compounds at 30 µM and control substances (DMSO) were added to the cells and incubated for further 20h. For Annexin V-staining cells were collected using Trypsin and resupended in 100 µl Annexin V binding buffer (BD Pharmingen). Subsequently, PE Annexin V (5 µL) (BD Pharmingen) and YO-PRO-3 (2 µL) (Invitrogen) was added and incubated for 15 min protected from light. After centrifugation (5 min, 1000 rpm) cells were again resupended in 500 µl Annexin V binding buffer and analyzed by FACS (Attune^®^ Acoustic Focusing Cytometer, Applied Biosystems^®^; detectors BL2/RL1). For analysis of apoptosis, cells were gated that are YO-PRO negative (R5) and Annexin V positive (R6).

1. Kempf SJ, Buratovic S, von Toerne C, Moertl S, Stenerlo ̈w B, et al. (2014) Ionising Radiation Immediately Impairs Synaptic Plasticity-Associated Cytoskeletal Signalling Pathways in HT22 Cells and in Mouse Brain: An In Vitro/In Vivo Comparison Study. PLoS ONE 9(10): e110464. doi:10.1371/journal.pone. 0110464
